# Hard Work and Hopefulness: A Mixed Methods Study of Music Students’ Status and Beliefs in Relation to Health, Wellbeing, and Success as They Enter Specialized Higher Education

**DOI:** 10.3389/fpsyg.2021.740775

**Published:** 2021-11-03

**Authors:** Dawn C. Rose, Carlo Sigrist, Elena Alessandri

**Affiliations:** School of Music, Lucerne University of Applied Sciences and Arts, Lucerne, Switzerland

**Keywords:** music, students, higher education, wellbeing, self-efficacy, self-determination, health, success

## Abstract

Using mixed methods, we explored new music students’ concepts of wellbeing and success and their current state of wellbeing at a university music department in Switzerland. Music performance is a competitive and achievement-oriented career. Research suggests musicians face vocation-specific challenges to physical health and mental wellbeing but has yet to investigate music students’ beliefs about wellbeing and success. With a self-report questionnaire (*n* = 99, Bachelor/Master students) we investigated new music students’ quality of life (WHO-5; WHOQoL-BREF) and self-efficacy (ASKU). Through qualitative workshops (17 groups, *n* = 5–8) we explored students’ understanding of the term “wellbeing,” and how this relates to “success.” Over half new music students (55%) believed the institution has 40–60% responsibility for their wellbeing. A simple linear regression showed that self-efficacy could predict better wellbeing, explaining 12% of the variance. Self-efficacy predicts wellbeing for new music students (β1 = 8.81, *p* = 0.001). The 17 flipcharts generated 121 inputs clustered into themes. Four themes solely described “wellbeing” (Health, Safety, Vitality, and Attitude) and four separately depict “success” (Achieving Objectives, Recognition, Career, and Financial Goods). Some themes intersected as elements of both constructs (Intersection: Relationships & Environment, Development, Happiness, Meaningfulness, Balance and Authenticity). Four further themes illustrated the relationship between the two (Reciprocity, Conditionality, Stability and Perspectivity). Music students believe responsibility for wellbeing is shared between themselves and their institution. As they scored low on both self-efficacy and wellbeing, these findings are an urgent call for action for school management and stakeholders of the music student population.

## Introduction

For musicians, there appears to be a paradox in the music and health narrative; on the one hand the mental and physiological challenges involved in trying to become or sustain a career as a musician can be detrimental to one’s physical health and psychological wellbeing (i.e., musculoskeletal problems, hearing damage, stress, and performance anxiety, see e.g., Vaag et al., 2014; Kenny and Ackermann, 2015). On the other hand, from a eudaimonic perspective (i.e., enjoying a meaningful vocation in life), musicians seem to enjoy a better sense of wellbeing in comparison to non-musicians (Ascenso et al., 2017, 2018). In order to know how best to promote and tailor wellbeing programs for musicians, it is important to understand the development over time of trigger issues for different aspects of musicianship, i.e., which psychological and physiological issues are “in play” for whom and at what stage. As shown in a longitudinal study, providing modules that offer psychoeducation tailored to music students’ issues does not necessarily improve health and wellbeing behaviors (Spahn et al., 2017).

As studies suggest it is the transition between student and professional life that is critical, personal and professional skills need to be adapted to self-support health and wellbeing, and notions of “success” may need to be re-framed as part of the reality of developing a portfolio career (Ascenso et al., 2016; López-Íñiguez and Bennett, 2020). The role of music institutions is not only to assess and guide playing abilities, but also to provide appropriate skills for the vocation (Wijsman and Ackermann, 2018). In terms of the development of the role of higher education (HE) music institutions, it is important to plan for and provide a framework of specialized support, especially considering the potential impact of the Covid-19 pandemic on musicians’ livelihoods (Elmer et al., 2020; Stanhope and Weinstein, 2020).

Few investigations however have documented the status of music students’ wellbeing at the start of their tertiary programs. In our previous study (Alessandri et al., 2020), we compared students enrolled on high performance study programs (sports and music) to students undertaking more typical university courses (e.g., sciences and humanities). Whilst wellbeing was compromised for all students compared to normative data, the personality trait of Emotional Stability and having an optimistic outlook contributed to predicting wellbeing in general for all students, with the personality trait Conscientiousness additionally contributing for sport and music students, and for music students only, perceived competence in their chosen vocation directly impacted their wellbeing. As the next step in understanding the state of play for our newly enrolled music students (what issues are already hiding in their music cases?), we present a cross-sectional mixed methods observational study of new music students at our institution. Specifically, this study focuses on new students during their first week in a Swiss HE music institution.

Our first aim was to document new music student wellbeing and perceived quality of life. Based on our previous study (Alessandri et al., 2020), we also included a measure of self-efficacy. Self-efficacy is a central tenet of self-determination theory and especially important in the study of music students as it directly relates to beliefs in one’s own capabilities to effect change (Bandura, 1977; De Castella and Byrne, 2015; Rose, 2016; Alessandri et al., 2020; Cohen and Panebianco, 2020). As noted by Morton et al. (2014), the ability to manage stressors is under-pinned by self-efficacy as difficulties encountered are seen as challenges to be overcome, rather than obstacles that undermine progress. We therefore surmised that higher levels of self-efficacy would predict higher levels of physical and psychological health (i.e., wellbeing) in music students.

As a further exploratory avenue of qualitative investigation, we asked our music students what they understood “wellbeing” to be in their context (i.e., training to be professional musicians), and expanded upon the notion of self-efficacy by asking students about “success” and how this might be linked to wellbeing for them. Our aim was to establish baseline information to understand the wellbeing status of our students, and how they are thinking about success and wellbeing as part of their vocational choice as they enter the first stages of their final training at our institution. With this information, our institution can then plan how best to support them through their transition into professional musicians, providing the adaptive skills necessary for life satisfaction and career longevity (López-Íñiguez and Bennett, 2020).

To summarize, our aims with this study were: (a) to document new music student wellbeing as the first stage of research in a longitudinal study using standardized measures that can be compared to other samples and populations, (b) to explore the role of self-efficacy in relation to the wellbeing of music students within the framework of self-determination theory, and (c) to consult our students about their beliefs with regard to wellbeing and success within the HE framework.

## Materials and Methods

This was a pilot study nested within a longitudinal research strategy to investigate music student wellbeing in specialist HE music institutions. Here we used a mixed method design with new students (i.e., in the first week of their first semester at our school). To provide further context, given the tremendous negative impact Covid-19 has had on HE students and the cultural sector in general (see e.g., Elmer et al., 2020; Cohen and Ginsborg, 2021): this study was conducted in Switzerland from 8th to 10th of September 2020. Social distancing and hygiene measures had been in place since March 2020 and during spring semester 2020 most of the university courses had to be held in a complete distance-learning-format. Our music school moved to new premises during summer 2020 and in combination with the covid-related restrictions, access to practice and rehearsal resources was limited. However, over summer and at the beginning of 2020’s autumn semester, some measures were loosened by the government providing a sense of hope (e.g., mixed format teaching was allowed, together with some smaller concerts, then larger events). Although the focus of the study was not related to the effects of Covid-19 on music students, we asked participants, “Are you currently concerned that the Covid-19 pandemic could have a negative impact on your musical activity?.” Just over half (*n* = 54, 55%) affirmed they were concerned about this matter. At the time of data gathering for this study, the gradual loosening of the restrictions may have instilled a sense of hopefulness in terms of professional and personal prospects for the students. Nevertheless, general travel restrictions were in place with conditions which may have negatively impacted concert-related travel and the large international student contingent.

### Procedure

Quantitative and qualitative data were collected from 8th to 10th September 2020 exclusively during a new 90-min introductory course on “Health and Wellbeing” offered to all new music major Bachelor and Master students during the university opening week. The course was mandatory for Bachelor students and non-mandatory for Master students. First, questionnaires (please see Supplementary Material 1 for the questionnaire text) were presented as hard copy forms in German language. Participants were then assured that the study had been granted ethical approval by the appropriate authorities and were provided with the Participant Information Sheet. The music students were then asked to provide demographic information, and then to answer the selected self-report measures. Participation was voluntary and options were offered, within that timeframe, for students to fill-out the questionnaire in privacy. After a short break, students were then invited to participate in a brainstorming session in groups of 5–8 students. Each group received flipcharts and colored markers to visualize their ideas. There was a brief presentation by each group at the end and participants were debriefed about the nature of the research. All Covid-19 related requirements such as mask-wearing and physical distancing as well as proper sanitization and hygiene were met during the sessions.

### Participants and Recruitment

All newly enrolled Bachelor students, for whom the Health and Wellbeing course is mandatory, chose to participate in the study (*n* = 89). However, six questionnaires were not completed sufficiently (<60% completion) to warrant inclusion. For Master students, for whom the course is non-mandatory, half the class attendees chose to participate (*n* = 17). Of these questionnaires, only one was not included due to non-completion. This led to a final sample of *N=99* for the survey data; 83 Bachelor and 16 Master music students.

### Qualitative Methods

Brainstorming techniques (Osborn, 1963) have been shown to be useful for idea generation within the higher education context (see review, Al-Samarraie and Hurmuzan, 2018). Seventeen groups of 5–8 students were asked, in a face-to-face setting, to brainstorm (i.e., discuss and illustrate) their definitions of (1) “wellbeing,” (2) “success,” and (3) to describe the relationship between the two constructs. The groups (which due to Covid-19 safety regulations were formed in advance by school management) were separated from each other, but not inaudibly. During sessions lasting 15–20-min, the groups produced handwritten flipcharts (see Supplementary Material 2 for a selection of these data). The sessions were facilitated by the final author who clarified the questions or tasks but did not moderate the sessions to minimize interference with process and group dynamics. The flipcharts were then independently and inductively coded by the second and the final authors in close alignment with guidelines for *Structured Content Analysis* (Kuckartz, 2019). Codes were compared and categorizations derived with main themes summarized in an iterative process, performed using MAXQDA 2020 (VERBI Software, 2019).

### Quantitative Measures

The survey included demographic variables to characterize the sample as well as the following self-report measures:

The WHO-5 is a brief (five item) generic rating scale of subjective wellbeing (Bech et al., 2003; Topp et al., 2015). The five statements are positively worded and scored using a 6-point Likert scale over the past two weeks. The Cronbach’s alpha coefficient reported for the WHO-5 is >0.80 (e.g., Garland et al., 2018).

The WHOQoL-BREF (WQB; Whoqol Group, 1998) is an abbreviated (26 item) version of the WHOQoL-100 which provides a Quality of Life (QoL) profile based on data from 18 countries. The manual defines QoL as *“an individuals*’ *perception of their position in life in the context of the culture and value systems in which they live and in relation to their goals, expectations, standards, and concerns”* (p. 3). Two separate items concern the individuals’ perception of a) their overall QoL and b) their general health. The remaining items produce scores for four domains (please see Supplementary Material 3 for details on domain concepts) described as Physical Health (7 items), Psychological Health (6 items), Social Relationships (3 items) and Environment (8 items). All items are rated using appropriate 5-point scales (i.e., *intensity*, *evaluation*, *satisfaction*, *capacity*, and *frequency*). The reliability of the domains is acceptable according to Cronbach alpha values (0.68, 0.75, 0.64, and 0.74 respectively, DeVellis, 2003).

The Self-Efficacy Scale – Short form (Allgemeine Selbstwirksamkeit Kurzskala (ASKU) in German, Beierlein et al., 2012) contains three items (related to self-reliance for situation-, problem-, and task-solving) scored using a 6-point Likert scale. The ASKU is a shortened and validated version of the 10-item General Self-Efficacy Scale by Schwarzer and Jerusalem (1995). Beierlein et al. (2013) provide reliability estimates using McDonald’s Omega values between 0.81 and 0.86, which they interpreted as sufficient.

The following section characterizes the students in terms of general demographics and information specific to music students.

## Results

### Data Preparation and Analyses

When assumptions for parametric analyses were met, Student *t* tests were used where appropriate (i.e., one sample *t*-test against normative/other published values). Non-parametric analyses were conducted where appropriate, for example due to the unequal sample (i.e., Welch’s t tests were used to compare between Bachelor and Master student groups, Delacre et al., 2017).

The choice to compare the data from this sample with three published data samples for the standardized tests (WHO-5 and WHOQol-BREF) was to provide contextual comparisons from the various perspectives. Students in higher education (HE) have been shown to have lower wellbeing than normative data in general, and some differences have been shown between students in HE whose focus is performance based in comparison to “other” types of study (Alessandri et al., 2020). The provision of contextual perspectives is especially important during extraordinary times such as those we are experiencing during the Covid-19 pandemic, where the burden educational institutions and the arts sector face (Marinoni et al., 2020; Spiro et al., 2021) reduces recruitment capacity for gathering directly comparative data.

Equal variances can be assumed unless otherwise reported. Alpha *p* value was adjusted for multiple comparisons using the Bonferroni method; alpha *p* = *p* < 0.005. Multiple regression analyses were conducted to ascertain the predictive value of variables of interest on wellbeing within this sample. Pearson’s bivariate correlational analyses (two-tailed) were conducted between measures. Where *n* and % are reported, the percentage data refers to the valid percent (i.e., the percentage is weighted according to missing values). Data was analyzed using the Statistical Package for Social Sciences (SPSS; v27, IBM).

### Quantitative Results

As this study focused on new intake students only, we compared the large intake of Bachelor and the small number of Master students using Welch’s *t*-test (which is robust against difference in group sizes). We expected significant differences in Age (and the related variables; “Years of Study” and “Average numbers of concerts performed per year (pre-Covid-19).” As expected, Master students were older, had spent more time studying their main instrument (14 rather than 11 years), and performed more concerts per year (prior to Covid-19) on average (between 11 and 40 concerts per year compared to between seven and ten) than Bachelor students (see Table 1). With regard to wellbeing measures, although a significant difference was found between student groups for the single item WQB-QoL with Master students experiencing worse QoL than Bachelor students (see Table 1), this result was not a robust finding once adjusted to the alpha *p* value for multiple comparisons. No other between-group differences were found in this sample. The general lack of difference between groups justified collapsing the data sets to combine Bachelor and Master students in their first semester at our institution.

**TABLE 1:** Demographic and descriptive information about the sample and between group statistics.

	Bachelor (*n* = 83)			Master (*n* = 16)			Welch’s t-test statistics
	Mean	SD	Range	Mean	SD	Range	Between groups
Age (years)	21.78	4.92	17–52	25.56	2.76	22–31	*t*(97) = −2.97, *p* = 0.004
Time per week doing side job (hours)	6.86	4.74	1–20	9.4	3.45	6–15	ns, *p* > 0.2
Total time studying main instrument (years)	11.16	4.17	2–23	14.06	4.51	3–23	*t*(97) = −2.52, *p* = 0.013
Amount of daily musical practice (hours per day)	2.77	1.37	1–7	3	1.24	1–6	ns, *p* > 0.5
Amount of daily other study (hours per day)	1.44	1.17	0–6	1.12	0.67	0–2	ns, *p* > 0.3
Number of concerts played per year (pre Covid-19)^a^	2.98	1.36	0–6	4.38	1.26	2–6	*t*(96) = −3.81, *p* < 0.001
Psychological issues in relation to musical activities^b^	2.61	0.61	1–4	2.54	0.66	1–3	ns, *p* > 0.6
Physical issues in relation to musical activities^b^	2.23	0.83	1–3	2	0.82	1–3	ns, *p* > 0.3
Continued practice despite physical discomfort^c^	3.52	1.54	1–5	3.5	1.41	1–5	ns, *p* > 0.9
Covid related anxiety about musical activity^d^	1.46	0.5	1–2	1.44	0.51	1–2	ns, *p* > 0.8
WQB QoL (Raw)	4.31	0.74	2–5	3.86	0.66	3–5	*t*(92) = 2.17, *p* = 0.034
WQB General Health (Raw)	3.69	0.78	2–5	3.87	0.74	2–5	ns, *p* > 0.4
WQB Physical Health (%)	74.45	15.38	21–100	72.14	16.98	36–100	ns, *p* > 0.6
WQB Psychological Health (%)	69.62	14.96	4–96	74.17	14.10	54–96	ns, *p* > 0.2
WQB Social Relationships (%)	75.22	17.57	33–100	76.92	17.73	42–100	ns, *p* > 0.7
WQB Environment (%)	78.29	14.56	39–100	75.0	12.99	47–94	ns, *p* > 0.4
WHO-5 (%)	61.82	15.56	12–84	60.57	21.56	24–96	ns, *p* > 0.7
ASKU (Raw)	3.75	0.68	1–5	3.73	0.57	1–5	ns, *p* > 0.9

*^*a*^Coded scale per year: 1 = one to three, 2 = four to six, 3 = seven to 10, 4 = 11 to 20, 5 = 21 to 39, 6 = 40+ concerts.*

*^*b*^Coded scale: 1 = currently, 2 = in the past, 3 = never.*

*^*c*^Code scale: 1 = Never, 2 ≤ once per month, 3 = 1–3 times per month, 4 = 1–3 times per week, 5 = almost daily.*

*^*d*^Covid Anxiety 1 = Yes, 2 = no.*

The final sample (*N* = 99; 83 Bachelor, 16 Master students) had a mean age of 22.39 (*SD* = 4.84, range 17–52). The sample was split evenly between males (*n* = 47, 48%) and females (*n* = 49, 50%) with three participants choosing not to describe their gender. Almost half (*n* = 46, 47%) had a side job for which they spent, on average, seven hours per week working (ranging from 1 to 20 hours per week). Instruments included Voice (*n* = 22, 22%), Guitar and Piano (*n* = 11, 11% each), Violin (*n* = 9, 9%), Drums (*n* = 5, 5%), Clarinet, Viola, Flute and Cello (*n* = 4, 4% each), Saxophone, Trumpet, French Horn, and Conducting (*n* = 3, 3% each), Double Bass, Electric Bass, Organ, Trombone and Oboe (*n* = 2, 2% each), and Choir Leading, Schwyzerörgeli, and Harp (*n* = 1, 1% each).

#### Descriptive Statistics

##### Student Self-Report of Instrument-Related Issues and Discomfort While Playing

We asked students whether they are currently and/or have in the past suffered from complaints that could be related to their musical activity physically and psychologically (forced choice; currently, in the past, never). Just under two thirds (*n* = 56, 61%) of the students reported currently and/or having suffered with playing related physical issues, and one third (*n* = 29, 33%) of students reported currently and/or having suffered with playing related psychological issues. Overall, *n* = 64 (68%) of student reported they had at some stage suffered with playing-related issues (either physical or psychological), *n* = 20 (21%) of these students reported having suffered with both types of issues, and *n* = 30 (32%) of students surveyed reported they had never suffered with playing-related physical or psychological issues.

Students were not asked to provide details on the nature of the experienced issues.

In this sample, *n* = 82, 85%) of music students reported that over the last six months they practiced on their main instrument, despite experiencing physical discomfort. Specifically, *n* = 39 (40%) reported this was something they did almost daily, *n* = 16 (17%) reported 1 to 3 times per week, *n* = 13 (13%) reported 1 to 3 times per month, *n* = 14 (14%) reported less than once per month. In the last six months, *n* = 15 (16%) reported that they never practiced on their main instrument while experiencing physical discomfort.

##### Student Beliefs About Ratio of Responsibility: Student and Music School

We asked students to rate the proportion (percentage) to which the music school carries responsibility for their own wellbeing using two slider scales (one for the individual, the other for the institution, indicating the total amount should be 100%, see Supplementary Material 1). Almost all of the music students surveyed (*n* = 82, 98%) believed that the music school was at least 20% responsible for their wellbeing.

Specifically, *n* = 27 (32%) of music students surveyed believed they themselves were 80% responsible for their own wellbeing, *n* = 26 (31%) believed they were 60% responsible, and *n* = 22 (26%) believed the responsibility was split equally between themselves and their institution (i.e., 50%/50%). No students believed their institution was more than 80% responsible for their wellbeing, but *n* = 5 (6%) of students believed the institution was 60% responsible for their wellbeing, and *n* = 2 (2%) believed their institution was 80% responsible for their wellbeing. Students were not asked to specify the areas of responsibility.

##### Components of Success Ranking

Students were asked to rank the relative importance of *practice*, *luck*, and *talent* for their success as a musician (forced choice between the three factors). Two thirds of students surveyed *n* = 53 (65%) ranked *practice* as the most important factor, almost a half (*n* = 35, 44%) ranked *talent* as the second most important factor, and almost a half (*n* = 56, 46%) ranked *luck* as the least important factor. The most popular ranked order was Practice, Talent, Luck (*n* = 32, 39%).

#### Inferential Statistics

##### WHO-5

Table 2 presents the results of the inferential analyses. Normative data for the WHO-5 should be based on the country of the participants [see Supplementary Table 2 in Topp et al. (2015) for country specific normative data]. In this study, Germany was the closest cultural match to German speaking area of Switzerland with a Mean percentile score of 66.3%. Therefore, this percentile score was used in statistical analysis to compare this sample with the normative data (one-sample Student *t* tests). Students in this study reported WHO-5% Mean score that was significantly lower than the Topp et al. (2015) normative data from Germany (*Mean difference* = −4.67, *CI* = −1.28 to −8.06).

**TABLE 2:** Comparison of WHO-5 and WHOQoL-BREF (WQB) wellbeing scores against study sample and comparative data (i.e., published normative, music and other student data).

	Study Sample	Topp et al. (2015) (Normative Data; German sample)		Baumann et al. (2014) (General HE Students)		Antonini Philippe et al. (2019) (Music HE Students)	
Variable	Mean (SD)	Mean (SD)	Statistic	Mean (SD)	Statistic	Mean (SD)	Statistic
WHO-5 (%)	61.63 (16.46)	66.3	*t*(92) = −4.66, *p* = 0.008				
WQB – Physical Health (Raw)	5.86 (2.49)	16.8 (2.6)	*t*(96) = −3.74, *p* < 0.001				
WQB - Physical Health (%)	74.09 (15.57)			72.90 (14.90)	ns, *p* > 0.4	69.46 (12.79)	*t*(96) = 2.93, *p* = 0.004.
WQB - Psychological Health (Raw)	15.25 (2.38)	15.7 (2.4)	ns, *p* = 0.07				
WQB - Psychological Health (%)	70.34 (14.85)			73.60 (14.40)	*t*(94) = −2.14, *p* = 0.35	69.09 (12.54)	ns, *p* > 0.4
WQB - Social Relationships (Raw)	16.07 (2.80)	14.4 (2.9)	*t*(89) = 1.67, *p* < 0.001				
WQB - Social Relationships (%)	75.46 (17.51)			74.00 (18.30)	ns, *p* > 0.4	77.02 (17.19)	ns, *p* > 0.4
WQB - Environment (Raw)	16.45 (2.28)	13.0 (2.3)	*t*(94) = 14.76, *p* < 0.001				
WQB - Environment (%)	77.77 (14.31)			74.00 (13.80)	*t*(93) = 2.55, *p* = 0.012	72.83 (15.64)	*t*(93) = 3.34, *p* = 0.001

##### WHOQoL-BREF

For the nearest geographical/cultural norms, the raw data^1^ from the German field trials were used (*n* = 2408, Skevington et al., 2004). Music students at our institution scored significantly lower than German norms for Physical Health (*Mean difference* = −0.95, *CI* = −0.44 to −1.45), but significantly higher than German norms for Social Relationships (*Mean difference* = 1.67, *CI* = 1.09–2.26) and Environment (*Mean difference* = 3.45, *CI* = 2.98–3.91). The sample from our institution did not significantly differ from German norms for Psychological Health.

To compare our data to general students^2^ in Western Europe, we used data from Baumann et al. (2014) who investigated first year students at the University of Luxembourg (*N* = 973), *Mean age* = 20.6 (*SD* = 3.2), range 18-44, 46% female. Our sample differed from Baumann et al. on two factors: percentile scores for Psychological Health were significantly lower for the students at our institution compared to general HE students (*Mean difference* = −3.26, *CI* = −0.24 to −6.29). However, the significance level of this statistic does not withstand adjustment for multiple comparisons. Our sample scored significantly higher for Environment (*Mean difference* = 3.77, *CI* = 0.83–6.7). Our sample did not significantly differ from general HE student scores for Physical Health and Social Relationships.

To compare our data to music students in Switzerland we used data published in Antonini Philippe et al. (2019). Their sample (*N* = 126) comprised music students (amateur and in higher education for music) in a French speaking area of Switzerland. Where possible, statistics were compared to the Higher Education subset of music students (*n* = 46). Music students at our institution scored higher than in Antonini Philippe et al. (2019) study on Physical Health (*Mean difference* = 4.63, *CI* = 1.49–7.77), and Environment (*Mean difference* = 4.94, *CI* = 2–7.87). Our sample did not significantly differ from scores at this comparable music school for Psychological Health and Social Relationships.

To summarize the significant results of the WQB measures, our sample scored lower than German norms and general HE students on Physical Health though higher than a comparable music school, higher than German norms for Social Relationships, and higher than German norms, general and music specific HE students for Environment.

##### Allgemeine Selbstwirksamkeit Kurzskala Measure of Generalized Self-Efficacy

Students at our institution scored a mean value of 3.77 (*SD* = 0.68) for the ASKU. This was significantly lower than the appropriate stratification of normative data for German residents, aged 18-35, who have completed 11 or more years of schooling (“*High*” *Norm mean* = 4.28, *t*(92) = −7.73, *p* < 0.001. Mean difference = −0.53, CI = −0.40 to 0.67).

##### Correlations

The WQB-Psychological Health and Physical Health factors were correlated with the WHO-5% (*r* = 0.75, *p* < 0.001, and *r* = 0.59, *p* < 0.001 respectively). In line with our previous study (Alessandri et al., 2020), the WHO-5% was considered the most parsimonious measure of wellbeing that accords with the WHO conceptual and holistic approach to wellbeing as a subjective life experience and not merely the absence of disease or infirmity (World Health Organisation [WHO], 1948).

##### Simple Linear Regression

A simple linear regression was carried out to test if self-efficacy significantly predicted wellbeing as conceptualized with the WHO-5%. The results of the regression indicated that the model explained 12.1% of the variance and that the model was significant, *F*(1, 89) = 12.31, *p* < 0.001. Self-efficacy significantly predicted wellbeing for new music students (β*1* = 9.60, *p* = 0.004). The same analysis was used to explore whether self-efficacy significantly predicted physical health indicated that the model explained 18.4% of the variance. The model was significant, *F*(1, 91) = 20.58, *p* < 0.001. Self-efficacy significantly predicted physical health for new music students (β*1* = 8.53, *p* < 0.001).

### Qualitative Findings

**FIGURE 1:**
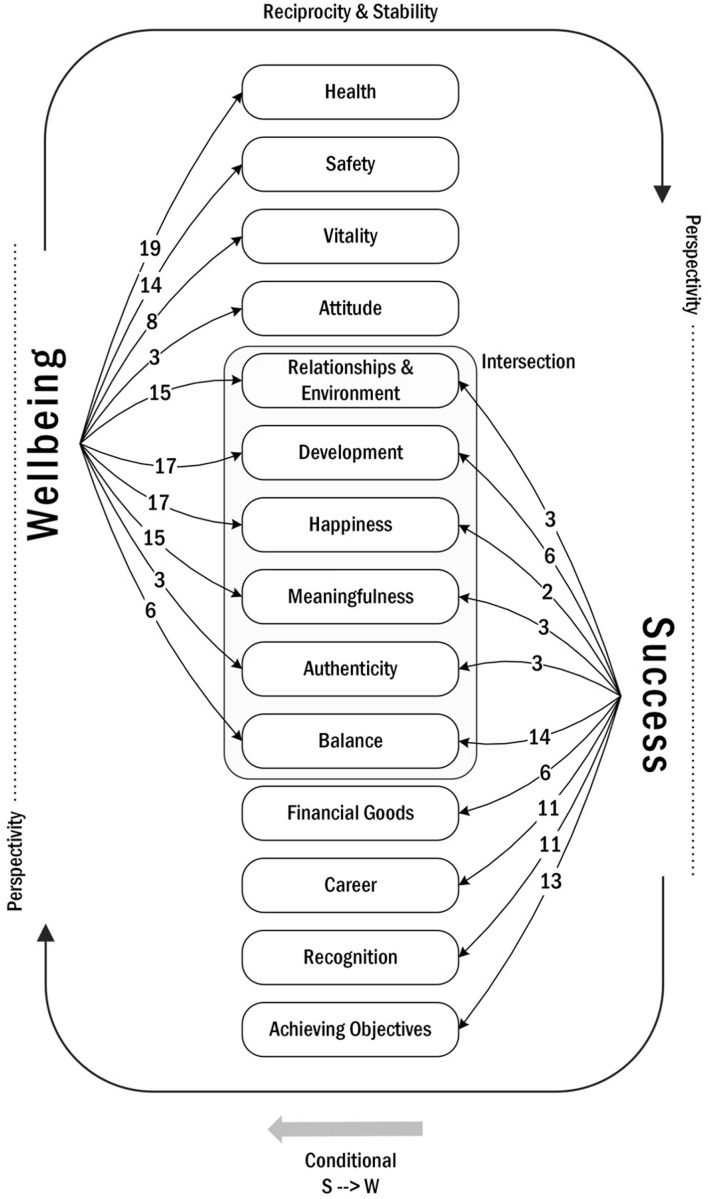
Visualization of themes emerged from the qualitative analysis of students’ flipcharts.

Over the 17 flipcharts (see Supplementary Material 2 for examples of raw data), students used a total of 121 inputs (i.e., keywords or short statements) to describe “wellbeing” and a total of 73 inputs to describe “success.” Students’ descriptions of “wellbeing” and “success” could be clustered in 15 themes. Four further themes emerged, that illustrate how students see the *relationship* between the two constructs. Figure 1 shows a summary visualization of the themes. “Wellbeing” and “success” emerged from students’ descriptions as being intimately interconnected: over one third of the descriptors used to describe “wellbeing” and “success” (*Relationships & Environment*, *Development*, *Happiness*, *Meaningfulness*, *Balance* and *Authenticity*) were identified as being elements of both constructs (*Intersection*). Four elements were described as belonging to “wellbeing” alone (*Health*, *Safety*, *Vitality*, and *Attitude*) and four to “success” alone (*Achieving Objectives*, *Recognition*, *Career*, and *Financial Goods*). “Wellbeing” and “success” as well as components thereof were described in general as influencing each other (*Reciprocity*). Students also tried to capture the *Conditional* relationships between the two constructs: they emphasized that while “wellbeing” and “success” are strongly connected, and yet neither of them is sufficient to achieve the other – although “wellbeing” is necessary precondition for “success” (S – > W). In two flipcharts, “wellbeing” and “success” were described as balancing each other out (*Stability*). Finally, students described “wellbeing” and “success,” with all their components, as being dependent on individual values, as well as cultural and societal norms and expectations (*Perspectivity*). Table 3 reports the full list of themes.

**TABLE 3:** Qualitative coding for elements of, and relationship between, wellbeing and success according to music students.

Theme	Frequency		Definition of theme
	Wellbeing	Success	
**Elements**			
Health	19	0	Physical and psychological health. Students often used the term “health” directly or variations of “being fit.” Physical pain and discomfort are also included here, along with conditions of sleep (or lack thereof) as a symptom associated with health.
Safety	14	0	Safety is either directly mentioned or aspects of it are thematized such as financial security, protection, being worry-free, and the fulfillment of basic needs.
Vitality	8	0	Inputs describe having energy and being active (i.e., doing something, or going for a walk), or lack thereof, such as being “exhausted.”
Attitude	3	0	Here instances of “acceptance” and “attitude” are clustered. Attitude is conceptualized as readiness to accept a situation, person, or reality.
Relationships & Environment	19	4	Terms that either describe social bonds (family, friends, social contacts) or assume functioning relationships (e.g., “constructive feedback,” “love”). This theme also includes terms that describe the social or daily environment (“life situations,” “relationship with the world,” “good food/drink”). One instance mentioned having a “good relationship with yourself.”
Development	17	6	Personal development including aspects of learning and discovery (“experience new things,” “learn something new,” and “develop yourself”).
Happiness	17	2	Short or long-lasting positive feelings, positive awareness of own emotional state, e.g., “to feel good,” “to be happy.” This theme also includes direct or indirect mentions of “peace” and “relaxation” (“calm,” “breathe,” “serenity”).
Meaningfulness	15	3	Having purpose and making meaning of one’s actions and activities including “motivation” and “feeling involved.” Also perceiving one’s work as a “vocation,” having objectives, and making an impact on other people (“to have an effect,” “to reach people”).
Authenticity	3	3	Feeling of identity and inner coherence (“authenticity,” “find your true self,” “resonate with your core self and with the world (body, spirit, soul)”). Constructs of “self-worth,” “self-realization,” and “freedom” are also included here.
Balance	6	14	Inner psychological balance (e.g., between Eu- and Distress) or balance between life areas (“work-life balance”). Instances of “free time,” “having time for yourself” as well as “being satisfied” are also encompassed, alongside “satisfaction” which is understood by the students as equation between expectations and reality.
Financial Goods	0	6	Relevant economic goods, like money, good payment, or material possessions.
Career	0	11	Work and career, including “performance,” “job” and in general having a chance to play in public and enjoying a “large audience.” This theme also includes inputs that focus on career status and quality of the work done, e.g., “career at professional level” or “a place in an orchestra.”
Recognition	0	11	Recognition, respect, appreciation, and esteem, e.g., perceived “status,” and peer acknowledgment “confirmation through colleagues.”
Achieving Objectives	0	13	Students often mention achieving personal or self-given objectives, i.e., “reaching goals.”
Perspectivity	2	9	Success and in two instances also wellbeing are viewed here as being subjective, “individual,” and dependent on situations, personal values, and expectations, including cultural and societal norms.
**Relationship**			
Reciprocity	10		Mutual influence between Wellbeing and Success (or components thereof), either generally expressed “success and wellbeing are strongly connected” or implying causality through arrows and words (e.g., “wellbeing leads to success” or “can influence each other.” In one instance this relationship between Wellbeing and Success was described as “symbiosis.”
Conditional	6		Necessity and sufficiency. Students affirm that despite the strong interconnection between Wellbeing and Success, alone neither would be sufficient to achieve the other. However, Wellbeing was described as necessary for Success.
Intersection	5		Wellbeing and Success are seen as overlapping (intersecting) sets. Success (and its components) was described as part of Wellbeing and reverse.
Stability	2		Wellbeing and Success balance each other out.

## Discussion

This cross-sectional study allows only a snap-shot view of the status and beliefs of new music students in relation to wellbeing and success as they enter the final stages of their music education. As surmised, self-efficacy predicted physical and psychological health/wellbeing. However, levels of self-efficacy were low, and we cannot be sure to what extent the Covid-19 situation impacted self-efficacy scores. In this respect it will be interesting to collect longitudinal data to see how those scores develop. Nevertheless, as students perceived a “shared responsibility” between themselves and the institution in terms of wellbeing, believed that practice is what matters the most in terms of their success, and yet weren’t confident they could shape their own future, these findings support calls for concrete action within higher education music schools.

Researchers have suggested a cultural shift is necessary to truly support students through this critical transition stage, and that a holistic approach is required, yet these aims may be at odds with the priorities of many institutions (Ascenso et al., 2016; Wijsman and Ackermann, 2018; Burland, 2020; López-Íñiguez and Bennett, 2020). Here we discuss how findings from this study can contribute to understanding the needs of our students in relation to the role of self-efficacy in transformative professionalism, the importance of environmental and social aspects of higher music education institutions, and the necessity to re-frame what constitutes “success,” and notions about playing through pain.

Self-efficacy has been linked to success in study and work, as well as good health and wellbeing in terms of motivating and maintaining behaviors, and appraising one’s own competencies to plan, act and achieve one’s desired goals (Bandura and Schunk, 1981; Dweck and Master, 2012). Recommendation to learn more about health-promoting behaviors has resulted in optional modules being offered in higher music education curricula due to the high prevalence of physical and psychological difficulties, though this approach only improves health-promoting behaviors in a minority of students (Spahn et al., 2017). However, the way a person regulates their behaviors may be an interaction between their own beliefs and actions, and their contextual environment. In this study for example, Bachelor and Master students appeared similar as they entered their new school of music, but future studies should also take care not to assume that Bachelor and Master students develop at the same rate depend on which variables are of interest. A recent study of musicians and self-efficacy in relation to the uptake of health-promoting behaviors (Cohen and Panebianco, 2020) showed that not only did self-efficacy mediate the influence of the personality traits of conscientiousness and extraversion in musicians, but also that students enrolled at a coastal university campus engaged in more physical activity than those whose campuses were further inland. This finding is in accordance with Bandura’s Social Cognitive Theory (Bandura, 1989) which posits that environment can either facilitate, or be perceived as barriers, be perceived as a barrier to motivations and behaviors that support wellbeing.

In our study, students scored higher than average on the Environment and the Social Relations domains of the WQB. Our sample specifically focused on new students, and furthermore, these students were the first intake of a new school of music building, specifically designed for musicians, including various spaces for music students to study, socialize and relax. The school is well positioned with a large lake and mountains nearby, and good public transport links. However, being accepted to a higher education music program usually requires years of cost-intense musical education and practice, which might imply a positive association between socioeconomic background and access to music education (Associated Board of the Royal Schools of Music [ABRSM], 2014; Burland, 2020). It will be important for future studies to further investigate the individual impact of contextual matters such as family, school, and local environment on students’ wellbeing.

A general study of Swiss student experiences during lock-down emphasized the importance of social relations in terms of wellbeing (Elmer et al., 2020). We see here some evidence that music students’ social networks were strengthened despite reduced physical and psychological wellbeing. We speculate that these new students either relied upon previously established support networks, or (less likely) identified and bonded with each other over their experience in a new environment. Considered in light of the three pillars of self-determination theory (i.e., relatedness, autonomy, and competence) the impact of social relations in music schools should not be underestimated, alongside the impact of contextual factors such as environment. We therefore suggest a holistic model of wellbeing should make use of local resources within and beyond the school and emphasize the design of social spaces to facilitate sustainable social interactions between students. As WQB does not deliver a descriptive answer to regular health and risk behaviors of music students, further research will be needed to better understand the nature and quality of students’ regular health and risk behaviors (e.g., habits in regards to exercise, diet, sleep, drug use and/or misuse of medication and other behaviors to cope with stressful situations) and their effect on musicians’ health and wellbeing as well as performance, as previous studies provide somewhat ambiguous results (Kreutz et al., 2008; Ginsborg et al., 2009; Perkins et al., 2017; Cruder et al., 2020).

Recent studies have suggested that musicians should now be considered as multi-professionals, but that the challenges associated with such a myriad skillset (i.e., struggling with enforced entrepreneurship, building, and running a small business, and having to take on other work to support themselves) is a problem much exacerbated in recent times (López-Íñiguez and Bennett, 2020; Spiro et al., 2021). To prepare music students for the realities of professional life, institutions need to move beyond prescriptive teaching and learning of repertoire, technique, and skills. In their investigation of a fellowship program with the Civic Orchestra of Chicago, Ascenso et al. (2016) found that when music students were immersed in a professional context, they extended their beliefs and behaviors beyond the comfort zone of conservatoire life. Students reported gaining perspective and improving their wellbeing by considering their roles as musicians and citizens. In addition to developing professional and social skills (i.e., time-management and networking), the students acquired confidence in their abilities to sustain a multi-faceted career in music. They felt like they had “something to say” (p. 161) and realized “there is so much more to music than just perfect performance” (p. 162). Rather than being directed to one goal, they could see there were multiple avenues available and felt more connected rather than disillusioned. Similarly, in their study reviewing the career profiles and opinions of successful cellists, López-Íñiguez and Bennett (2020) found that reframing of the concept of success was seen as an essential way to overcome hierarchical and narrow perceptions of achievement in the classical music world and avoid a crisis of identity later in their careers. Moreover, developing ways to uphold ethical behaviors (e.g., being able to challenge bullying, sexism and racism) was seen as a way to avoid the repetition of default teaching modes (i.e., the master-apprentice model of instrumental learning, Burwell, 2016) and instead foster artistic as well as social and professional diversity.

The insights gained from the qualitative part of our study suggest the students may be ahead of the institutions in these matters. Students saw a clear connection between wellbeing and success and described success not just in terms of tangible or external outputs like concerts, earning or recognition, but also in terms of psychological and life balance, social relationships, and finding meaning in their own doing. They spoke of authenticity and integration in their communities that reflect the studies described above and demonstrate how musicians now embrace being agents of social change, rather than accepting the *status quo*. The interconnection between success and wellbeing—as described by our students—suggests an implicit understanding of wellbeing in line with current models of eudemonic wellbeing (Ryff and Singer, 2008; Huta, 2013). In our students’ views, feelings of happiness, safety and inner balance are enmeshed with achievement and excellence: they not only influence each other but perhaps even define what “success” ought to be, including concepts such as personal development, meaningfulness and authenticity. In addition to the wellbeing elements to be found in eudemonic models of wellbeing like PERMA (Seligman, 2011), in a few instances our students described a readiness to accept circumstances as they are (i.e., acceptance) as a component of wellbeing. Contentment and acceptance have been recently embedded in wellbeing models rooted in existential positive psychology, like the mature happiness model (Wong and Bowers, 2018). During the time of the Covid-19 pandemic, the mature happiness model was shown to be a better predictor of general psychological distress than the PERMA model (Carreno et al., 2021). This construct of attitude as an ability to accept and find personal growth in negative or non-optimal aspects of living emphasizes once more the importance of a holistic approach to wellbeing and a redefinition of the notions of success that HE music institutions nurture among students.

Nevertheless, the issue of playing-related pain remains in our sample. The high proportion of students reporting instrument-related physical issues and the low level of physical health resonate as a call for help in students’ flipcharts, in which physical health, including freedom from pain, are identified as a main component of their wellbeing. This is in line with the findings of Wijsman and Ackermann (2018) who suggest that whilst the development of performance-based medical disorders began in childhood (for many musicians), practice habits exacerbate this, contributing to chronic conditions in later life. At the point of entry into tertiary music education, most music students face an increased risk of injury due to increased practice and performance demands. They suggest a cultural shift is required, including a settings-based approach to health literacy as low levels of health literacy among teachers can perpetuate the cycle. The authors further propose that the success of such a program will depend on contextual factors such as *relevance*, *accessibility*, *legitimacy of knowledge*, and the *practical implementation of injury prevention strategies*. This translational approach to developing learning cultures is recognized in the WHO Shanghai Declaration (World Health Organisation [WHO], 2017) which states that health literacy must be an integral part of the skills and competencies developed over a lifetime and incorporated into the educational curriculum. As pointed out by Stanhope and Weinstein (2020), musculoskeletal disorders are the most common and costly compensation claims made by musicians. They suggest that although medical advice is typically to cease doing whatever is causing the pain, musicians may require a more nuanced approach to treatment. This is because: (a) they are more likely to experience pain of greater intensity than any tissue damage indicates (compared to population norms) owing to both neurological differences and higher prevalence of sleep problems and psychological distress, and (b) taking time off has serious consequences for musicians such as financial jeopardy, stigma, and loss of reputation (letting colleagues down), as well as a potential loss of identity, and supportive social connections. The multi-center study being carried out by Cruder et al. (2020) will undoubtedly provide valuable insights for institutions considering how best to implement prevention and treatment programs.

In terms of our study, to protect the anonymity of the participants, we did not ask for specific details about the nature of the playing related pains, and/or other physical and psychological conditions our students reported. Nevertheless, that they reported such high incidences suggests that the sacrifice involved in “playing through the pain” (a concept also prevalent in elite sports) may be related to the conflict between talent myths and self-determination theory; how can one overcome the lack of magic dust? Only by practicing more. These conflicts and paradoxes are inherent within the system of musical learning; from motivating beginners (practice makes perfect narratives) to honing the skills of the advanced students (how can you be better than everyone else). As we move forward with considering how best to support our students, the potential of a “living curriculum” approach (Johnsson and Hager, 2008) that emphasizes curiosity and creative autonomy would seem to capitalize in the intrinsic motivation for lifelong learning apparent in musicians (Rose, 2016; Burland and Davidson, 2017). Moreover, empowering not just the students as stakeholders, but also the whole staff in terms of a culture of learning that underpins good practice to embed health and wellbeing in music education is necessary from an institutional perspective (Riva et al., 2020). The “process of discovering” forms part of a growth mindset (in contrast to a fixed mindset focused on the achievement of externally devised goals thereby undermining self-efficacy, Dweck and Master, 2012; De Castella and Byrne, 2015), that can be applied in both directions (i.e., staff and students). López-Íñiguez and Bennett (2020) suggest four key areas for change: 1. enabling musicians to become *agents of learning*, 2. reducing reliance on the master-apprentice model of instrumental learning, 3. fostering ideas around multi-professional nature of life as a musician, and 4. promoting the social consciousness of students (musicians as citizens) to encourage perspective related wellbeing. Future directions include an ongoing international study comparing music schools and music students across stages and types of study to understand the changes throughout the undergraduate process and into the post graduate stages.

## Data Availability Statement

The raw data supporting the conclusions of this article will be made available by the authors, without undue reservation.

## Ethics Statement

This study, involving human participants, was reviewed and approved by the Ethics Committee of Lucerne University of Applied Sciences and Arts, Lucerne, Switzerland. The participants provided written informed consent prior to taking part in the study.

## Author Contributions

DR and EA designed the study. DR analysed the quantitative data. EA conducted the qualitative workshops; EA and CS analysed the qualitative data. All authors contributed to the manuscript and approved the final version.

## Conflict of Interest

The authors declare that the research was conducted in the absence of any commercial or financial relationships that could be construed as a potential conflict of interest.

## Publisher’s Note

All claims expressed in this article are solely those of the authors and do not necessarily represent those of their affiliated organizations, or those of the publisher, the editors and the reviewers. Any product that may be evaluated in this article, or claim that may be made by its manufacturer, is not guaranteed or endorsed by the publisher.

## Footnotes

1 Percentile data are not available for Swiss norms.

2 Fields of study described as Science & Technology, Law & Finance, and Social Sciences.
