# Supplementary material for: Hard Work and Hopefulness: A Mixed Methods Study of Music Students’ Status and Beliefs in Relation to Health, Wellbeing, and Success as They Enter Specialized Higher Education
Source: Front Psychol. 2021 Nov 3;12:740775. doi: 10.3389/fpsyg.2021.740775 (PMC8596639; doi:10.3389/fpsyg.2021.740775)
Supplement: Supplementary file 1 [file Presentation_1.pdf]

Supplementary Material 1: Text version of the survey.

Introduction (text not provided here)

My gender:

- female
- male
- other
- prefer not to answer

My age in years:

My nationality: (Drop down list of nations provided).

Do you have one or more part-time jobs alongside your studies?
(For example: an internship or job in journalism, sound engineering, administration, supermarket/shop sales assistant, or working in hospitality (bar, restaurant or hotels etc.).

- Yes, I have a job alongside my studies.
- No, I am not working while I am studying.

How many hours per week (on average) do you work in your job, in addition to studying?

In which sector(s) do you carry out this work? (For example, sales, hospitality etc.).

I am currently enrolled in:

- Bachelor
- Master in Performance (Instrumental/Vocal)
- Master in Music Pedagogy (Instrumental/Vocal)
- Master in Theory, Composition, or Research

Current semester in my study program: (Drop down list 1-16 provided)

My main instrument is:

How many years have you been playing your main instrument?

My main style of music is:

- ▢ Classical
- ▢ Jazz
- ▢ Folk Music
- ▢ Church Music
- ▢ Contemporary Art Music

Approximately how many hours a day do you practice on your main instrument (including vocals)?

How many hours per day do you invest in other study-related activities (e.g. theory, composition, analysis, mental training, physical exercises etc.)?

Approximately how many concerts did you play **per year** in the last 5 years (before the Corona pandemic)?

Please slide the markers (left or right) to the percentages that most closely reflect the degree of responsibility you feel for your wellbeing during your studies of music, and for what proportion should the HSLU M (i.e. your Music Institution) be responsible.
There are no right or wrong answers, but try to make your answer total 100%!

	Both Equal<br>0 10 20 30 40 50 60 70 80 90 100
Student	
Music Institute	

Health and Wellbeing
Please tick in the table below whether you are currently and/or have in the past suffered from complaints that could be related to your musical activity.

I suffer and/or have suffered from the following complaints...

	Currently	In the past	Never
Psychological discomfort (for example: symptoms of depression, anxiety…)	▢	▢	▢
Physical discomfort (for example: muscle aches, joint pains or other pains, cramps…)	▢	▢	▢

Over the last 6 months: How often, approximately, did you practice on your main instrument, despite physical discomfort?

- ○ Never
- ○ Less than once a month
- ○ 1 to 3 times per month
- ○ 1 to 3 times per week
- ○ Almost daily

Are you currently concerned that the Covid-19 pandemic could have a negative impact on your musical activity?

- ○ Yes
- ○ No

Which strategies and behaviour patterns help you to cope with study-related physical and psychological stress, such as physical complaints, performance-related anxieties, time management, etc? (Keywords are enough, open text box provided).

Please read each question, think about how you felt in the last two weeks and tick the answer that best suits you.

	Very poor	Poor	Neither poor nor good	Good	Very good
How would you rate your quality of life?	○	○	○	○	○

	Very dissatisfied	Dissatisfied	Neither satisfied nor dissatisfied	Satisfied	Very satisfied
How satisfied are you with your health?	○	○	○	○	○

The following questions are about how much you have experienced certain things in the last two weeks.

	Not at all	A little	A moderate amount	Very much	An extreme amount
To what extent do you feel that physical pain prevents you from doing what you need to do?	○	○	○	○	○
How much do you need any medical treatment to function in your daily life?	○	○	○	○	○
How much do you enjoy life?	○	○	○	○	○
To what extent do you feel your life to be meaningful?	○	○	○	○	○
How well are you able to concentrate? (	○	○	○	○	○
How safe do you feel in your daily life?	○	○	○	○	○
How healthy is your physical environment?	○	○	○	○	○

The following questions ask about how completely you experienced, or were able to do certain things in the last two weeks.

	Not at all	A little	Moderately	Mostly	Completely
Do you have enough energy for everyday life?	○	○	○	○	○
Are you able to accept your bodily appearance?	○	○	○	○	○
Have you enough money to meet your needs?	○	○	○	○	○
How available to you is the information that you need in your day-to-day life?	○	○	○	○	○
To what extent do you have the opportunity for leisure activities?	○	○	○	○	○

	Very poor	Poor	Neither poor nor good	Good	Very good
How well are you able to get around?	○	○	○	○	○

The following questions ask you to say how good or satisfied you have felt about various aspects of your life over the last two weeks.

	Very dissatisfied	Dissatisfied	Neither satisfied nor dissatisfied	Satisfied	Very satisfied
How satisfied are you with your sleep?	○	○	○	○	○
How satisfied are you with your ability to perform your daily living activities?	○	○	○	○	○
How satisfied are you with your capacity for work?	○	○	○	○	○
How satisfied are you with yourself?	○	○	○	○	○
How satisfied are you with your personal relationships?	○	○	○	○	○
How satisfied are you with your sex life?	○	○	○	○	○
How satisfied are you with the support you get from your friends?	○	○	○	○	○
How satisfied are you with the conditions of your living place?	○	○	○	○	○
How satisfied are you with your access to health services?	○	○	○	○	○
How satisfied are you with your transport?	○	○	○	○	○

The following question refers to how often you have felt or experienced certain things in the last two weeks.

	Never	Seldom	Quite often	Often	Always
How often do you have negative feelings such as blue mood, despair, anxiety, depression?	○	○	○	○	○

The following statements may more or less apply to you.
To what extent it applies to you personally.

	Doesn't apply at all	Applies a bit	Applies somewhat	Applies mostly	Applies completely
I can rely on my abilities in difficult situations.	○	○	○	○	○
I am able to solve most problems on my own.	○	○	○	○	○
I can usually solve even challenging and complex tasks well.	○	○	○	○	○

The following questions consider the extent to which students believe they can influence their musical talent themselves.

There are no right or wrong answers. We are only interested in your opinion.
Please indicate the extent to which you agree with each of the statements below.

	Strongly disagree	Disagree	Slightly disagree	Slightly agree	Agree	Strongly agree
My musical talent is something about me that I personally can't change very much.	○	○	○	○	○	○
I believe that I have the ability to change my basic musical talent considerably over time.	○	○	○	○	○	○
I don't think that I personally can do much to increase my musical talent.	○	○	○	○	○	○
No matter how much musical talent I currently have, I believe I am capable of changing it significantly.	○	○	○	○	○	○

Please rank the following terms in order of their relevance to your musical and professional success (1 = most important, 2 = middle, 3 = least important).
To do this, click on the respective term and drag it to the correct position while holding down the mouse button. There are no right or wrong answers.

Talent
Luck (and/or Coincidence)
Practicing (Studying and Working)

Finally, we would like to know more about how your general wellbeing has been over the last two weeks. For each statement, please mark the answer that you think best describes how you felt. Please take notice of the order of answers in this last set of questions.

In the last two weeks...

	All the time	Most of the time	More than half of the time	Less than half of the time	Some of the time	At no time
…I have felt cheerful and in good spirits	○	○	○	○	○	○
…I have felt calm and relaxed	○	○	○	○	○	○
…I have felt active and vigorous	○	○	○	○	○	○
…I woke up feeling fresh and rested	○	○	○	○	○	○
…My daily life has been filled with things that interest me	○	○	○	○	○	○

Debrief (text not provided here)
