# Supplementary material for: Hard Work and Hopefulness: A Mixed Methods Study of Music Students’ Status and Beliefs in Relation to Health, Wellbeing, and Success as They Enter Specialized Higher Education
Source: Front Psychol. 2021 Nov 3;12:740775. doi: 10.3389/fpsyg.2021.740775 (PMC8596639; doi:10.3389/fpsyg.2021.740775)
Supplement: Supplementary file 2 [file Image_1.pdf]

**Supplementary Materials 2**

Selection of four flipcharts as examples of qualitative data collection.

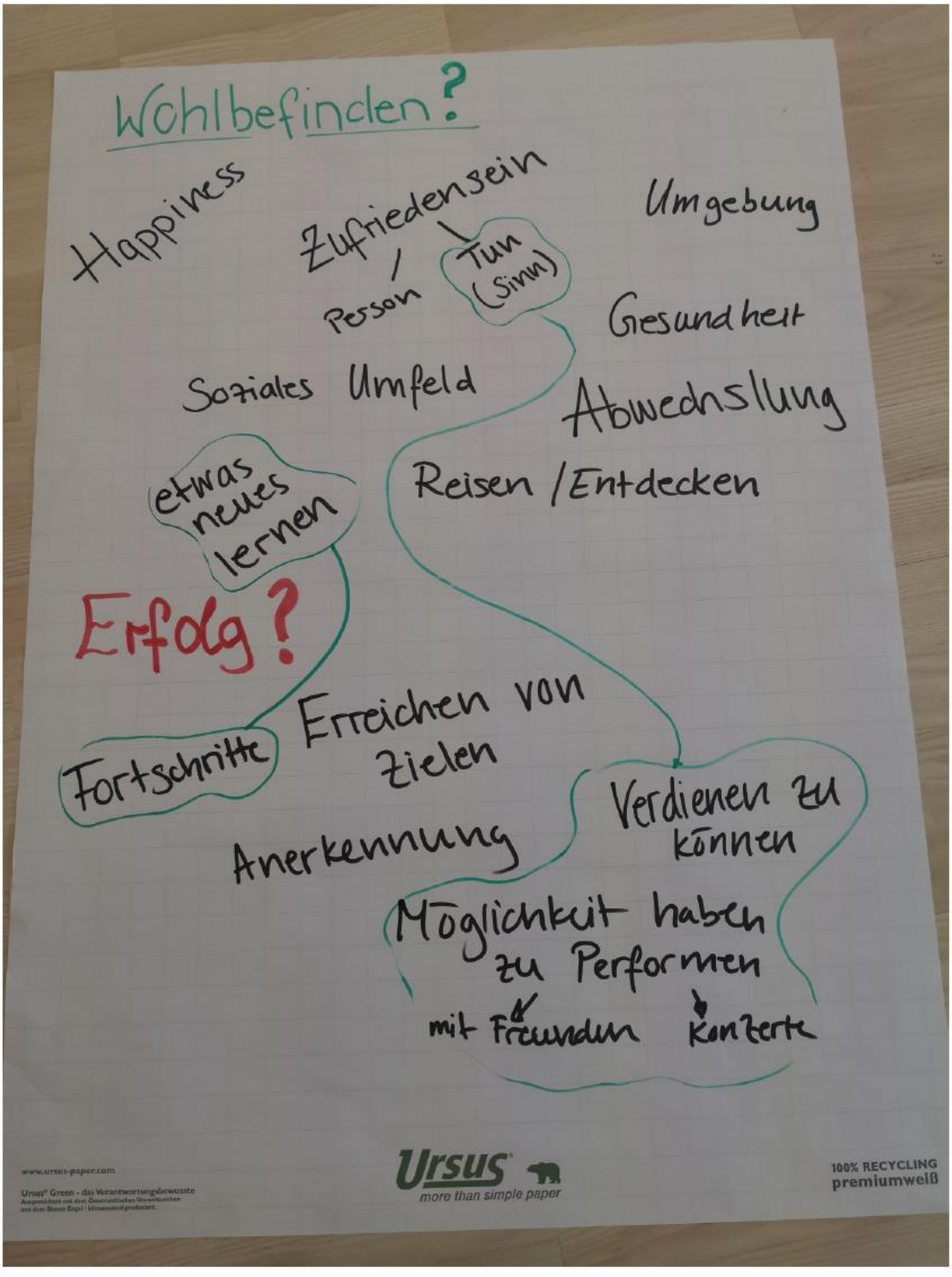

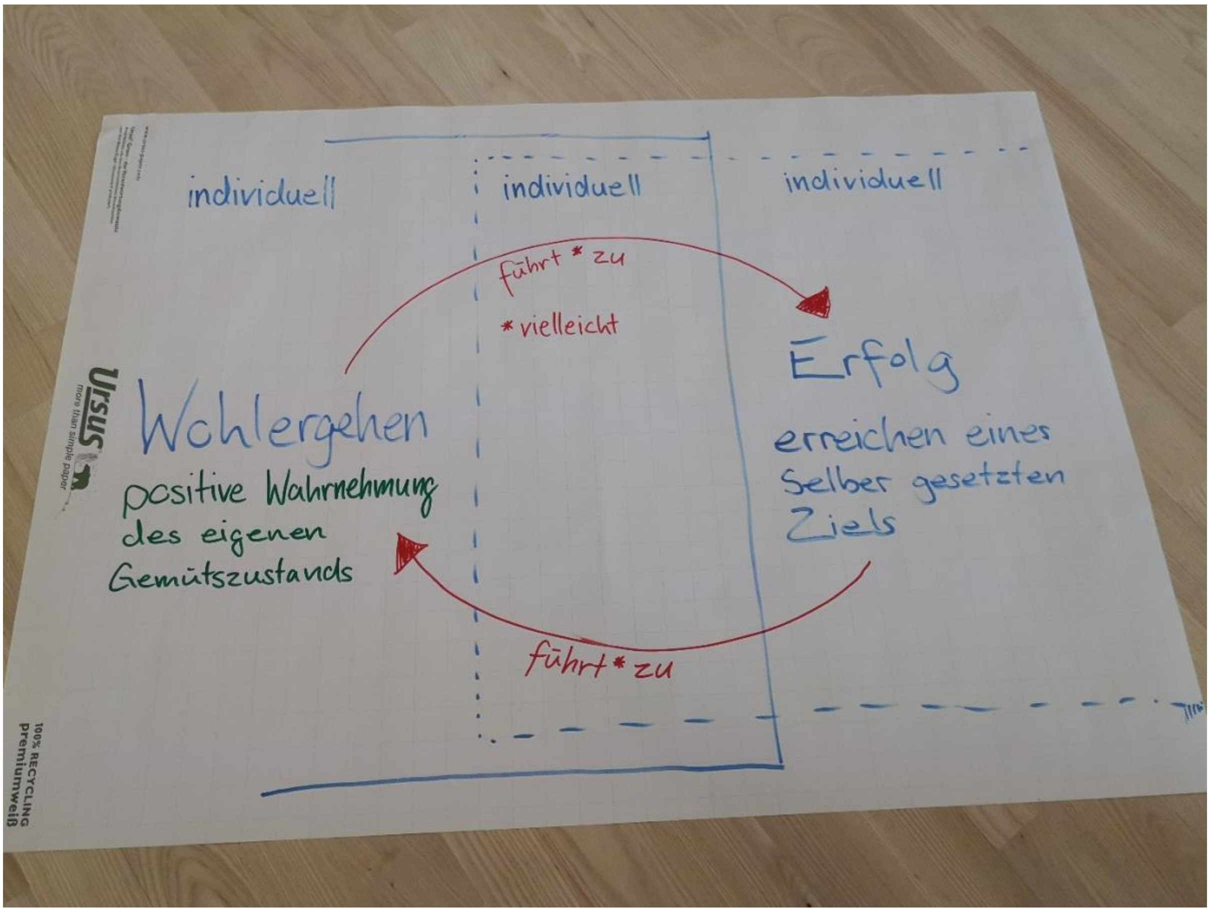
individuell
individuell
individuell
führt * zu
* vielleicht
Erfolg
Wohlergehen
erreichen eines
Selber gesetzten
Ziels
positive Wahrnehmung
des eigenen
Gemütszustands
führt * zu

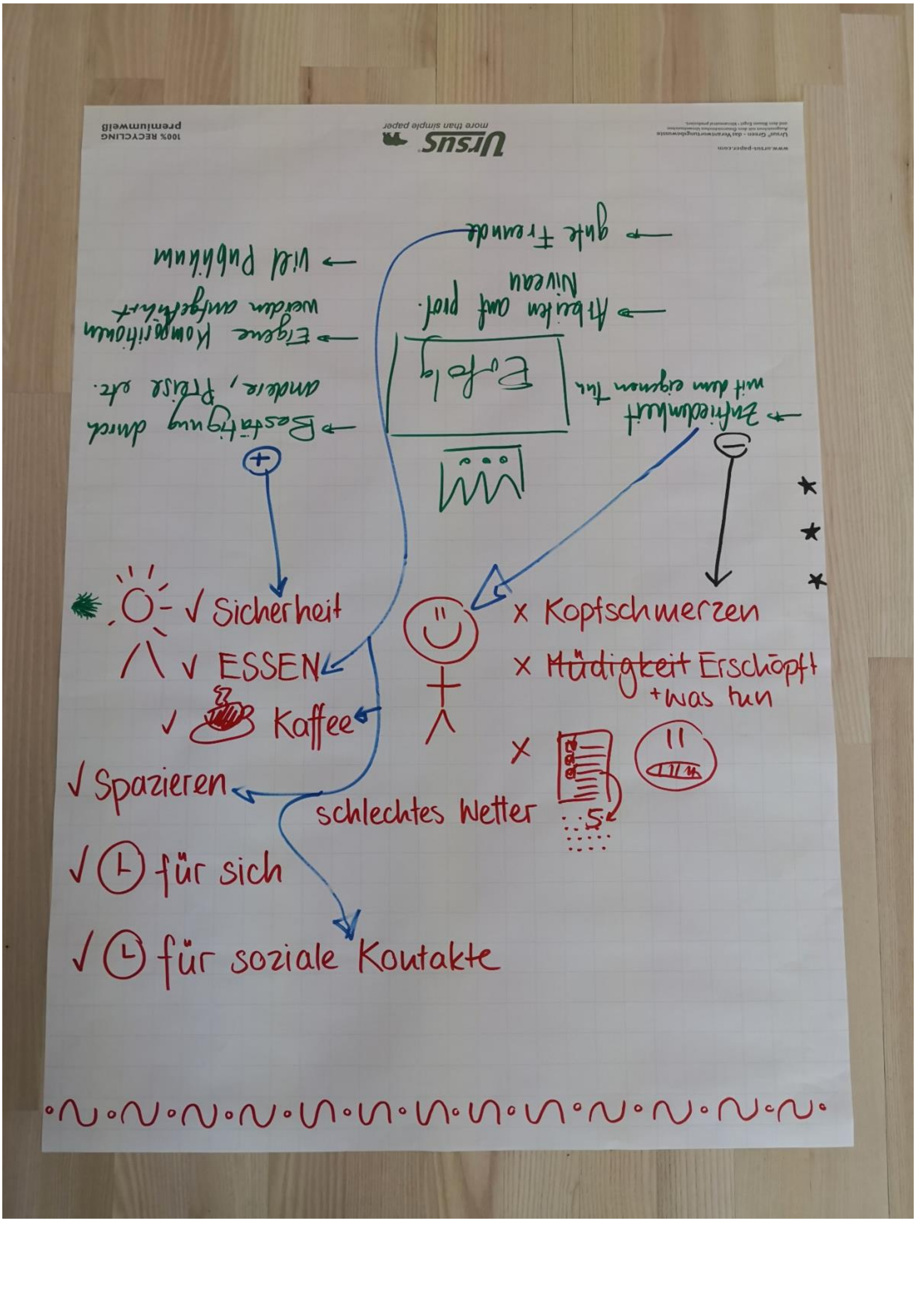
Ursus
more than simple paper
100% RECYCLING
premiumweiß
gute Freunde
viel Publikum
Arbeiten auf prof. Niveau
Eigene Kompositionen werden aufgeführt
Erfolg
Bestätigung durch andere, Preise etc.
Zufriedenheit mit dem eigenen Tun
Sicherheit
ESSEN
Kaffee
Spazieren
für sich
für soziale Kontakte
Kopfschmerzen
Müdigkeit Erschöpft
+was tun
schlechtes Wetter
5

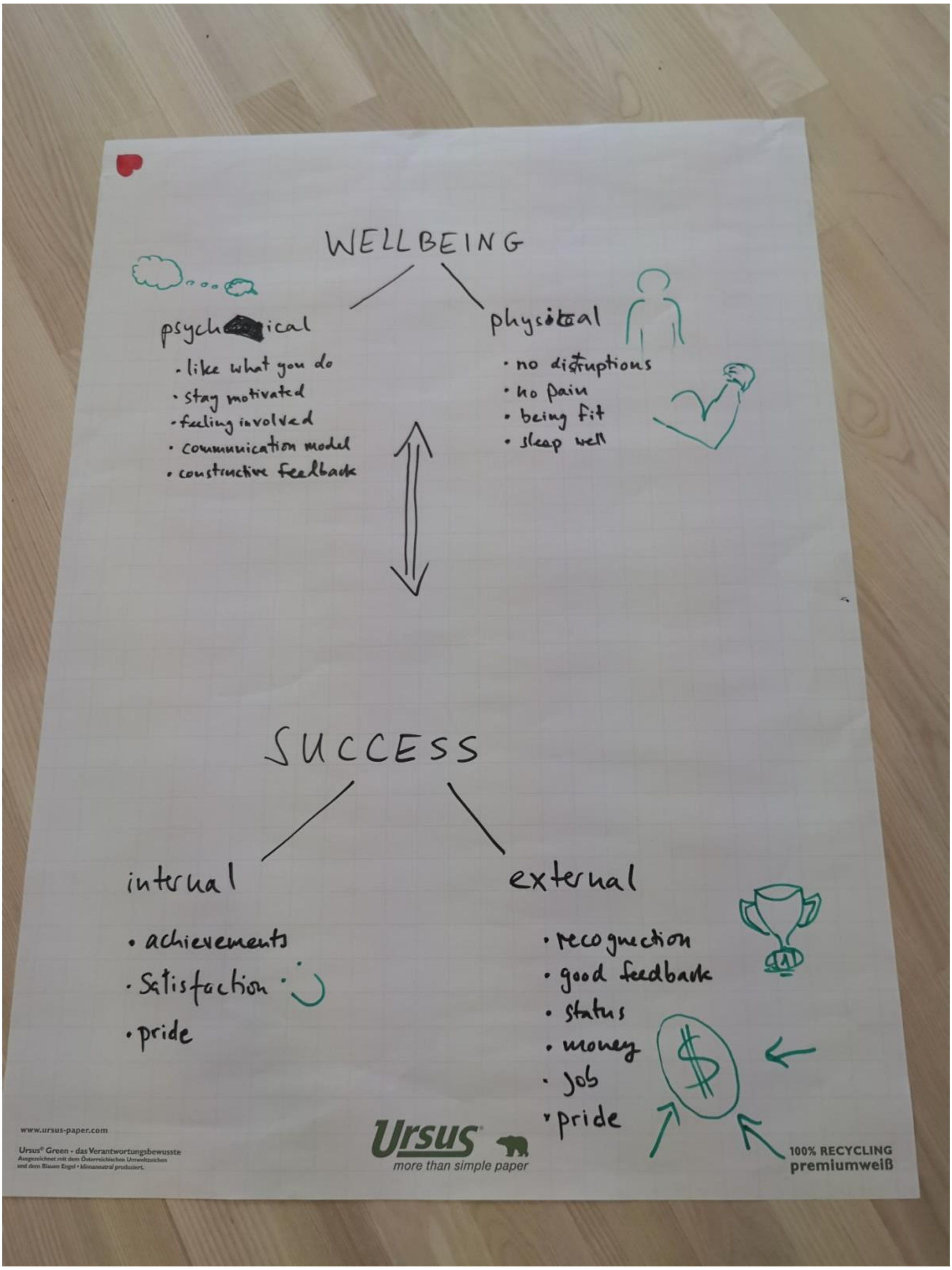
WELLBEING
psych[redacted]ical
• like what you do
• stay motivated
• feeling involved
• communication model
• constructive feedback
physical
• no distruptions
• no pain
• being fit
• sleap well
SUCCESS
internal
• achievements
• Satisfaction
• pride
external
• recognection
• good feedback
• status
• money
• job
• pride
www.ursus-paper.com
Ursus® Green - das Verantwortungsbewusste
Ursus
more than simple paper
100% RECYCLING
premiumweiß
