# Supplementary material for: Hard Work and Hopefulness: A Mixed Methods Study of Music Students’ Status and Beliefs in Relation to Health, Wellbeing, and Success as They Enter Specialized Higher Education
Source: Front Psychol. 2021 Nov 3;12:740775. doi: 10.3389/fpsyg.2021.740775 (PMC8596639; doi:10.3389/fpsyg.2021.740775)
Supplement: Supplementary file 3 [file Table_1.docx]

**Supplementary Material**

**S1.** The four factors refer to the individuals’ perception about their quality of life for each specific domain.

| **WHOQoL- BREF Factors** | **Facets incorporated in the domain** |
| --- | --- |
| 1. Physical Health | Activities of daily living, dependence on medical substances and medical aids, Energy and fatigue, Mobility, Pain and discomfort, Sleep and rest, Work capacity. |
| 2. Psychological | Bodily image and appearance, Negative feelings, Positive feelings, Self-esteem, Spirituality / Religion / Personal beliefs, Thinking, learning, memory and concentration. |
| 3. Social relationships | Personal relationships, Social support, Sexual activity. |
| 4. Environment | Financial resources, Freedom, physical safety and security, Health and social care: accessibility and quality, Home environment, Opportunities for acquiring new information and skills, Participation in and opportunities for recreation / leisure activities, Physical environment (pollution, noise, traffic, climate), Transport. |
